# RNA sequencing dataset characterizing transcriptomic responses to dietary changes in *Caenorhabditis elegans*

**DOI:** 10.1016/j.dib.2019.104006

**Published:** 2019-05-24

**Authors:** Scott T. Schumacker, Chloe A.M. Chidester, Raymond A. Enke, Matthew R. Marcello

**Affiliations:** a Department of Biology, Pace University, USA; b Department of Biology, James Madison University, USA; c Center for Genome & Metagenome Studies, James Madison University, USA

## Abstract

Transcriptome analysis using next generation sequencing (NGS) technology provides the capability to understand global changes in gene expression throughout a range of tissue samples. The nematode *Caenorhabditis elegans (C. elegans)* is a well-established genetic system used for analyzing a number of biological processes. *C. elegans* are a bacteria-eating soil nematode, and changes in bacterial diet have been shown to cause a number of physiological and molecular changes. Here we used Illumina RNA sequencing (RNA-seq) analysis to characterize the mRNA transcriptome of mixed *C. elegans* populations fed differing strains of bacteria to further understand dietary changes at the molecular level. Raw FASTQ files for the RNA-seq libraries are deposited in the NCBI Sequence Read Archive (SRA) and have been assigned BioProject accession PRJNA412551.

**Table:** Specifications Table

Subject area	*Biology*
More specific subject area	*Biochemistry, Genetics and Molecular Biology (General); Bioinformatics*
Type of data	*Table, text file, graph, figure*
How data was acquired	*RNA sequencing, Illumina NextSeq 500*
Data format	*FASTQ*
Experimental factors	*Extraction of total RNA from Caenorhabditis elegans fed E. coli OP50 or E. coli HB101 diets*
Experimental features	*Sequencing of polyadenylated mRNA followed by bioinformatics analysis for transcript analysis and variance assessment*
Data source location	New York, United States, Pace University; Cold Spring Harbor, New York, Cold Spring Harbor Laboratory
Data accessibility	The nucleotide sequences of raw reads were submitted to NCBI's Sequence Read Archive through the BioProject PRJNA412551 (https://www.ncbi.nlm.nih.gov/bioproject/PRJNA412551/)
Related research article	MacNeil, L. T., Watson, E., Arda, H. E., Zhu, L. J. & Walhout, A. J. M. Diet-induced developmental acceleration independent of TOR and insulin in *C. elegans*. *Cell***153,** 240–252 (2013).

**Table:** 

**Value of the Data** • These datasets will be valuable to the *C. elegans* research community for characterizing global changes in gene expression between environmental conditions. • These transcriptome datasets may be used to identify differentially expressed genes after dietary changes in *C. elegans.* • This bioinformatics analysis pipeline exclusively using open access tools to ensure sequence quality and robust eukaryotic transcriptome analysis. • This bioinformatics alignment-free pipeline reduces the time of analysis as well as required computing power which may be beneficial for some users, particularly in an undergraduate course setting.

## 1 Data

**Fig. 1:**
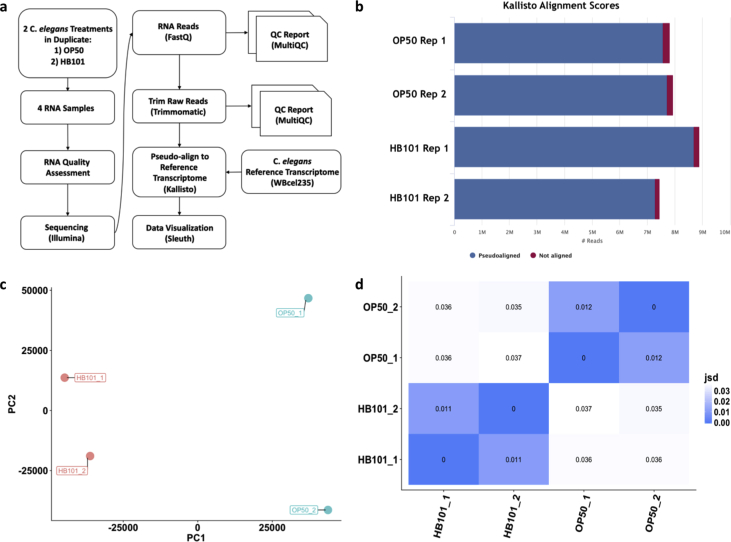
**Bioinformatics pipeline, assessment of read mapping and sample variance**. (a) Flowchart overview of the RNA-seq experiment. (b) Per sample summary of Kallisto pseudo-alignment of RNA-seq reads to *C. elegans* WBcel235 reference transcriptome. Number of reads are plotted on the x-axis is in millions (M). Additional details about the alignment are listed in Table 1. (c) Principal Component Analysis (PCA) Biplot of experimental sample variance. (d) Heat map analysis of experimental samples variance. [Key: Jensen Shannon Divergence (jsd) = similarity between samples; 0 = identical (blue); 1 = no overlap (white)].

Changes in diet can have profound effects on gene expression, especially genes encoding metabolic enzymes in the nematode *Caenorhabditis elegans* [1]. *C. elegans* is a well-established genetic system used for analyzing a number of biological processes. *C. elegans* are bacteria-eating soil nematodes, and changes in bacterial diet have been shown to cause a number of physiological and molecular changes [2]. Lifespan, fertility, and developmental rate have all been reported to change in response to diet [1], [2], [3], [4]. The data reported here analyze the transcriptional response after *C. elegans* are switched from a diet of *E. coli* OP50 to a high carbohydrate diet of *E. coli* HB101 [5]. Similar experiments have been conducted comparing animals fed *E. coli* HT115 and *Comamonas* DA187 [2]. To the author's knowledge, this is the first published data set detailing the transcriptome-wide expression changes comparing *E. coli* OP50 and HB101 diets. These analyses were conducted using Illumina mRNA-seq in tandem with a bioinformatics pipeline exclusively using open access tools to ensure sequence quality and robust eukaryotic transcriptome analysis (Fig. 1). The experiment described here is part of an on going NSF-funded project hosted by the Cold Spring Harbor Laboratory, DNA Learning Center (CSHL DNALC) focused on incorporating RNA-seq analysis into undergraduate education (http://www.rnaseqforthenextgeneration.org).

## 2 Experimental design, materials, and methods

### 2.1 *C. elegans* feeding

Recently starved *C. elegans* populations fed *E. coli* OP50 were transferred to either a fresh *E. coli* OP50 (control) or *E. coli* HB101 (experimental) diet. Four plates per condition were cultured for five days at 20 °C. Mixed *C. elegans* populations from each plate were collected, combined, and packed via centrifugation into a 15 ml polypropylene tube for RNA extraction.

### 2.2 RNA preparation and sequencing

**Fig. 2:**
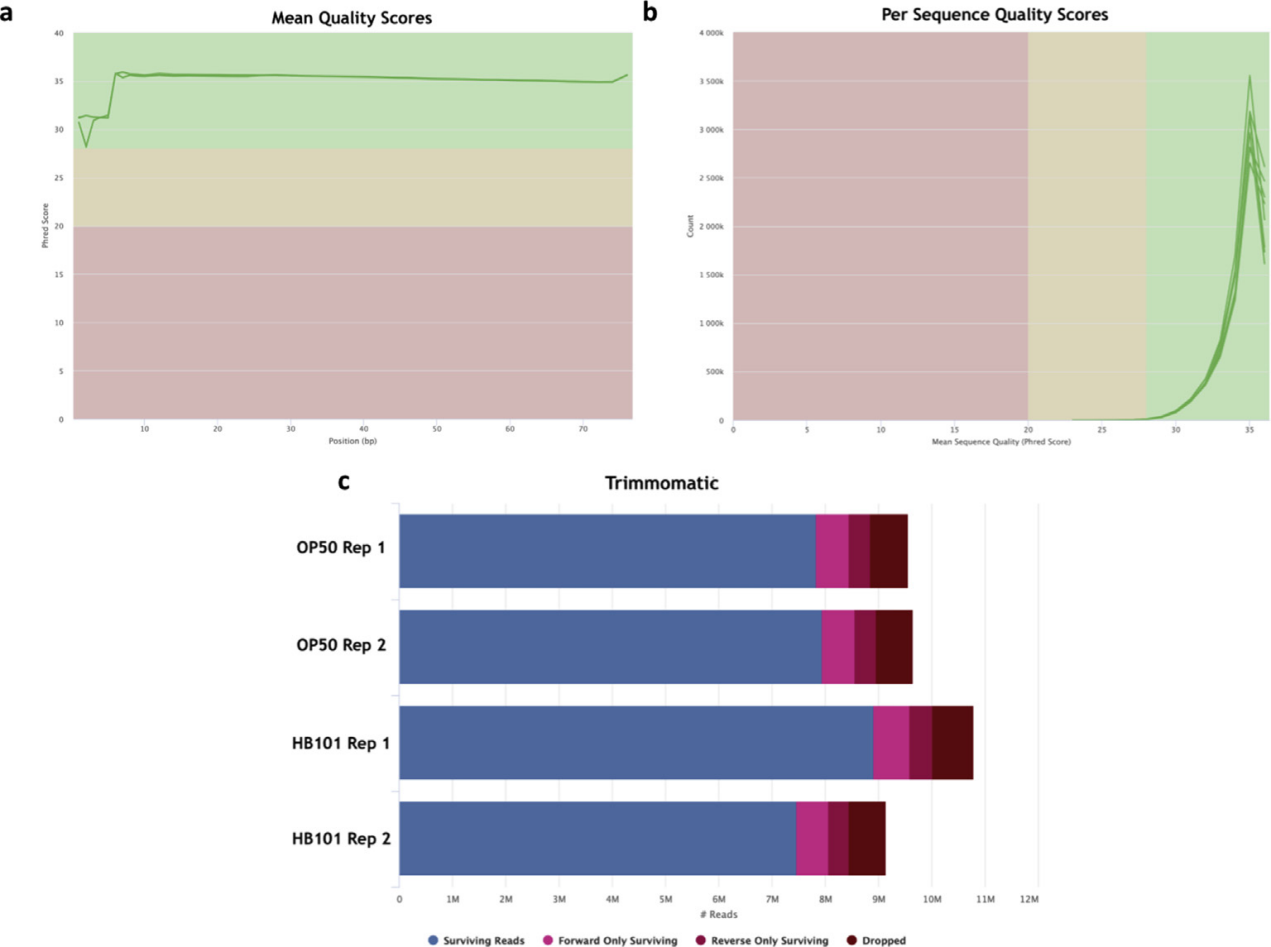
**FastQC and MultiQC quality assessment of unfiltered FASTQ data**. MultiQC summary plot of FastQC analysis demonstrate the RNA-seq read distribution of average per base (a) and per sequence (b) quality scores for each experimental sample file. (c) MultiQC summary plot of Trimmomatic filtering results (see Code Availability 1–3 for details of FastQC, Trimmomatic, and MultiQC software respectively).

Total RNA was extracted from mixed *C. elegans* populations using TRIzol reagent per the manufacturer's instructions (Invitrogen) [6]. Samples chosen for characterization of global mRNA expression were submitted to the CSHL DNA Sequencing Center for Bioanalyzer quality control analysis using a 2100 Bioanalyzer (Agilent). All submitted samples had RNA integrity number (RIN) > 8. Illumina stranded TrueSeq cDNA libraries were constructed using poly dT enrichment for each of the four samples in biological duplicate according to the manufacturer's protocol. The resulting average size of the cDNA libraries was approximately 300 bp. Libraries for the eight cDNA samples were sequenced using the Illumina NextSeq 500 sequencing platform yielding 9.1–10.8 million 75 bp paired end sequence reads per sample (Fig. 2c).

### 2.3 Sequence QC, filtering, and alignment

Fig. 1a demonstrates our experimental overview including the bioinformatics pipeline applied to our sequencing reads. Quality of individual FASTQ sequence files was evaluated using FastQC analysis (see Code Availability 1), Each FASTQ file was filtered using Trimmomatic [7] to remove minority truncated and low-quality reads (see Code Availability 2). Following Trimmomatic, filtered files were rerun through FastQC and summary per sequence and per base quality plots were created using the MultiQC program (see Code Availability 3). Fig. 2a–b demonstrates that all FASTQ sequencing files have an average per base and per sequence Phred score >28, a conventional threshold denoting high quality base calls. Fig. 2c and Table 1 demonstrate that the vast majority of sequencing reads were of suitable quality for downstream analysis. High quality reads were pseudo-aligned to the *C. elegans* WBcel235 reference transcriptome index using Kallisto [7] (see Code Availability 4). The percentage of aligned reads ranged from 96.8 to 97.7% (Table 1; Fig. 1b). Further quality validation of the data set was assessed using mapped reads from each sample. FastQC, Trimmomatic, MultiQC, and Kallisto transcript indexing and quantification were completed using applications hosted within the CyVerse Discovery Environment, a freely available cyberinfrastructure (https://www.cyverse.org/; see Code Availability 1–4).

**Table 1:** RNA-seq read statistics.

Sample name	Sequencer	Read length (bp)	psuedoaligned reads (in millions)	Uniquely mapped reads (%)
OP50 1	Illumina NextSeq 500	2 × 75	7.6	96.8
OP50 2	Illumina NextSeq 500	2 × 75	7.7	97.2
HB101 1	Illumina NextSeq 500	2 × 75	8.7	97.7
HB101 2	Illumina NextSeq 500	2 × 75	7.3	97.6

### 2.4 Transcriptome data analysis

Statistical analysis and visualization of global expression data between samples was quantified at the transcript level using the RStudio package Sleuth [8] (see Code Availability 5). Sleuth builds on traditional count-based methods of transcript quantification by applying improved estimates of transcripts and gene abundances [8]. In this analysis, Sleuth was used to assess variance between sample groups and sample replicates using principle component analysis (PCA) and distance matrix analysis. These experiments demonstrate the overall quality of our sample collection, library preparation, and sequencing (Fig. 1c–d).

### 2.5 Code availability

The following software and versions were used for quality control and data analysis as described in the main text:

1. FastQC, version 0.11.5 application was used within CyVerse Discovery Environment for quality analysis of raw FASTQ NGS data: http://www.bioinformatics.babraham.ac.uk/projects/fastqc/
2. Trimmomatic, version programmable-0.36 application was used within CyVerse Discovery Environment for trimming and filtering raw reads assuring read length and quality. Trim settings were SLIDINGWINDOW:4:20, LEADING:20, TRAILING:20, MINLEN:50: http://www.usadellab.org/cms/?page=trimmomatic
3. The MultiQC application was used within CyVerse Discovery Environment to create data plots summarizing FastQC, Trimmomatic, and Kallisto outputs: https://multiqc.info/
4. Kallisto, version 0.42.3 application was used within CyVerse Discovery Environment to create a reference transcriptome index and for the pseudo-alignment process: https://pachterlab.github.io/kallisto/.
5. Sleuth, a statistical model and RStudio package was used for sample quality analysis as well as normalization and visualization of differential gene expression analysis output: https://pachterlab.github.io/sleuth/about.

All walkthroughs and scripts used for quality assessment and data analysis in this analysis are available at: https://github.com/enkera/Enkera-Marcello-scidata2018-Celegans-rnaseq-diet.

## 3 Technical validation

### 3.1 Quality control-RNA integrity

Quality of total RNA fractions was assessed using an Agilent 2100 Bioanalyzer to calculate a RIN. The RIN algorithm determines the RNA quality of the samples with the highest quality having a score of 10. Conventional to NGS analysis, only RNA samples with a RIN >8 were used for sequencing analysis.

### 3.2 RNA-seq raw data quality

FastQC and MultiQC per base and per sequence quality analysis demonstrates mean Phred quality scores are well within the acceptable range for downstream analysis (Fig. 2a–b). After Trimmomatic filtering, FASTQ files contained 7.5–8.9 million high quality reads (Fig. 2c). 96.8–97.7% of these trimmed reads were successfully mapped to the *C. elegans* WBcel235 transcriptome assembly (Fig. 1b, Table 1).

### 3.3 Usage notes

The bioinformatics pipeline applied to our data set outlined in Fig. 1a uses a specific collection of freely available, open access research grade tools. FastQC, Trimmomatic, MultiQC, and Kallisto transcript indexing and quantification were completed using applications hosted within the CyVerse Discovery Environment and do not require any scripting. Statistical analysis and data visualization of transcript expression among samples was quantified at the transcript level using the RStudio package Sleuth [8]. These analyses however, are interchangeable with many other currently available tools. Our raw FASTQ data can be aligned to any available *C. elegans* reference genome or transcriptome using a variety of aligners. Aligned reads in the form of bam files can be viewed as intuitive BigWig density plots using popular genome browser such as the UCSC Genome Browser [9], the Ensembl Browser [10], or the Broad Institute's Integrative Genome Viewer (IGV) [11], [12]. Our transcript expression analysis was carried out using the Sleuth statistical model and RStudio data visualization package; however other publicly available packages such as edgeR [13] or Ballgown [14] can be used assuming that the reads were mapped to a reference genome rather than a reference transcriptome. Our currently presented alignment-free pipeline reduces the time of analysis as well as required computing power which may be beneficial for some users, particularly in an undergraduate course setting [7], [8].

Our data set will be useful for a variety of studies investigating transcriptional response to environmental changes in *C. elegans*. The simplicity of our experimental design set as well as the materials required for reproduction and/or further experimentation makes these data particularly useful for exposure of undergraduate students to RNA-seq transcriptome analysis; however, the further analysis would be strengthened by additional samples. It is possible that the exposure to dietary changes could modify the life cycle of the *C. elegans* and affect the age distribution of the population, thus confounding data analysis. In this case most of the detected changes could be due to this difference in life cycle and not due to the diet specifically. Additional data analysis modules available on the RNA-seq for the Next Generation website hosted by the Cold Spring Harbor Laboratory DNA Learning Center can be applied to further study the data set presented here as well as data gathered from other RNA-seq for the Next Generation and derivative studies [15], [16], [17], [18], [19], [20].

There are several considerations that must be accounted for when using these data for downstream analysis. First, RNAs were extracted from a mixed population of *C. elegans*. Therefore, resulting downstream analysis will be representative of heterogeneous mixtures of different aged animals. Second, cDNA libraries were prepared using a poly dT primer, thus the data set is representative of only polyadenylated mRNA transcripts and does not represent a subset of non-coding RNA or other non-polyadenylated cellular transcripts. Additionally, usage of poly dT priming introduces a bias towards overrepresentation of 3' ends of transcripts, particularly in the case of large transcripts. Finally, the quantity of sequenced and mapped reads per sample in this analysis (Table 1; Fig. 2c) is sufficient for robust differential expression analysis, however, is below the conventional threshold for thorough differential mRNA isoform analysis [21]. Taking these considerations into account, these data will be a useful resource for the *C. elegans* research community to investigate changes in gene expression that take place environmental and dietary changes.

## Funding

This work was supported by the National Institutes of Health [#1 R15 EY028725-01A]; the National Science Foundation [#1821657]; the Burroughs Wellcome Fund [#1017506]; and the JMU 4-VA Office.
